# Supplementary material for: Complexome profiling of the Chlamydomonas psb28 mutant reveals TEF5 as an early PSII assembly factor
Source: Plant Cell. 2025 Mar 17;37(6):koaf055. doi: 10.1093/plcell/koaf055 (PMC12164590; doi:10.1093/plcell/koaf055)
Supplement: koaf055_Supplementary_Data [file koaf055_supplementary_data.zip › psb28_interactive.zip!/plots/intensity/qualityPlots/peptides/_Cre15.g642050.t1.2.html]

### Cre15.g642050.t1.2

Trivial name:   
SeqCoverage [median]: 1.20  
Mass: 145.22 kDa  
  
 

| Sequence | MissedCleavage | Mass [Da] | wtIntensity | muIntensity | Score |
| --- | --- | --- | --- | --- | --- |
| MNALLAVAAAATDACLAR | 0 | 1744.90 | 1438.62 | 0.00 | 50.30 |
|  |  |  | 1438.6 | 0.0 |  |
